# Vitamin D Requirements for the Future—Lessons Learned and Charting a Path Forward

**DOI:** 10.3390/nu10050533

**Published:** 2018-04-25

**Authors:** Kevin D. Cashman

**Affiliations:** Cork Centre for Vitamin D and Nutrition Research, School of Food and Nutritional Sciences, University College Cork, T12 Y337 Cork, Ireland; k.cashman@ucc.ie; Tel.: +353-21-4901317

**Keywords:** vitamin D requirements, Dietary Reference Values, individual participant data

## Abstract

Estimates of dietary requirements for vitamin D or Dietary Reference Values (DRV) are crucial from a public health perspective in providing a framework for prevention of vitamin D deficiency and optimizing vitamin D status of individuals. While these important public health policy instruments were developed with the evidence-base and data available at the time, there are some issues that need to be clarified or considered in future iterations of DRV for vitamin D. This is important as it will allow for more fine-tuned and truer estimates of the dietary requirements for vitamin D and thus provide for more population protection. The present review will overview some of the confusion that has arisen in relation to the application and/or interpretation of the definitions of the Estimated Average Requirement (EAR) and Recommended Dietary Allowance (RDA). It will also highlight some of the clarifications needed and, in particular, how utilization of a new approach in terms of using individual participant-level data (IPD), over and beyond aggregated data, from randomised controlled trials with vitamin D may have a key role in generating these more fine-tuned and truer estimates, which is of importance as we move towards the next iteration of vitamin D DRVs.

## 1. Introduction

An enormous body of research in relation to various aspects of vitamin D and health over the last two decades has been instrumental in informing the estimates of dietary requirements for vitamin D that have emerged since 2011. While in North America the terminology used to describe the distribution of dietary requirements is Dietary Reference Intakes (DRI), its equivalent in Europe is Dietary Reference Values (DRV) [1]; DRV is an abbreviation I will, for ease, use throughout this review to represent both DRI and DRV, unless otherwise specified. These recent DRV are crucial from a public health perspective in providing a framework for prevention of vitamin D deficiency and optimizing vitamin D status of individuals [2]. In particular, DRV underpin the core goal of dietary assessment, which is to determine if the nutrient intakes of an individual or group are meeting the needs of that individual or group. While these are essential public health policy instruments developed with the evidence-base and data available at the time, there are some issues that need to be clarified or considered in future iterations of vitamin D DRV. This is important as it will allow for more fine-tuned and truer estimates of the dietary requirements for vitamin D and thus provide for more population protection. In particular, utilization of a new approach in terms of using individual participant-level data, over and beyond aggregated data, from randomised controlled trials (RCTs) with vitamin D may have a key role in generating these more fine-tuned and truer estimates. Consideration and clarification of some of the issues around DRV derivation for vitamin D will certainly represent ‘changing times for vitamin D and health’ in terms of dietary vitamin D requirements. The present review will highlight some of the clarifications needed and the potential of the newer approach to analyze data as we move towards the next iteration of DRV for vitamin D.

## 2. Lack of Clarity and Consistency of Definition May Be an Achilles’ Heel within Recent Vitamin D DRV Exercises

In scientific research, meaningful definitions are essential for comparability and reproducibility [3]. Furthermore, it has been suggested that to take effective public health measures, solid monitoring and evaluation programmes are necessary, for which the definition of the concept must be clear [3]. Within the context of DRV for vitamin D, defining one’s terms is also a key step.

The Institute of Medicine (IOM) in the US and the European Food Safety Authority (EFSA) both have published excellent overviews, or guides, to nutrient requirements in general [4,5]. The IOM’s DRI and EFSA’s DRV, both include an (Estimated) Average Requirement (EAR/AR), Recommended Dietary Allowance (RDA in the DRI) or Population Reference Intakes (PRI in the DRV), Adequate Intake (AI), and Tolerable Upper Intake Level (UL) (see Table 1). Of the four values in common within both sets of DRVs, the EAR and RDA (or their equivalents) deserve particular attention in light of their key role in ensuring nutritional adequacy of groups and individuals, respectively. The definitions provided by IOM and EFSA for their constituent DRI and DRV, respectively, are provided in Table 1 [4,5,6,7]. Despite this, however, the interpretation and application of these definitions has led to confusion and debate internationally which lessens the public health impact of some of these vitamin D DRVs. The following will overview a number of areas where clarification of the definitions, and their interpretation and application, would benefit future iterations of the vitamin D DRVs:

### 2.1. The Average Daily Nutrient Intake Level

**Figure 1:**
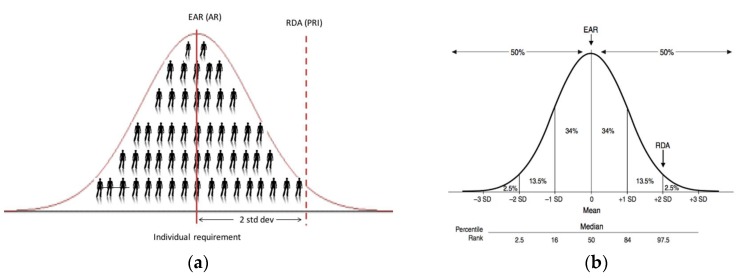
(**a**) The placement of (Estimated) Average Requirement [EAR(AR)] and Recommended Dietary Allowance (Population Reference Intakes) [RDA(PRI)] on the distribution of nutrient requirements within a population, as per the Institute of Medicine and European Food Safety Authority, and (**b**) and normal requirement distribution of hypothetical nutrient including percentile rank (taken from [4]).

Even the simple word ‘average’ within the IOM’s definitions of EAR and RDA can lead to a level of confusion. In the context of the EAR, it is ‘the average daily nutrient intake level that is estimated to meet the requirements of half of the healthy individuals in a particular life stage and gender group’ [4,6]. Thus, the fact that it is an intake intended to meet requirements of half of the healthy individuals (i.e., 50%; see Figure 1) puts the word ‘average’ in the title. The use of the word ‘average’ within the EAR definition proper, as well as that of the RDA (covering the needs of 97.5% of the healthy individuals) (see Table 1), relates to the level of vitamin D intake each day, on average over a period of time (e.g., a week or longer), which is estimated to meet the requirements. Thus, this ‘average’ over the period of time within an individual takes account of expected variability in the intake of vitamin D in a person’s diet arising from his/her food choice and consumption patterns.

There can be confusion around the application or interpretation of the ‘average intake’ element of the RDA definition. In general, RDAs, and their international equivalents, are intended for ensuring nutritional adequacy of individuals more so than groups. EFSA, in their 2010 opinion, have suggested that the PRI (their RDA equivalent, see Table 1) is an intake level that covers the requirement of 97–98% of all individuals when requirements of the group have a normal distribution (see Figure 1b); it should therefore not be used as a cut-point for assessing nutrient intakes of groups because a certain overestimation of the proportion of the group at risk of inadequacy would result [5]. Despite this, the Nordic Council of Ministers (NORDEN), for example, when setting recommendations for their populations have suggested that the Recommended Intake (RI; their RDA equivalent) is appropriate for an average intake of a group expressed per day over a period of one week or more [8]. Possibly unintentionally, the IOM in the manner by which they derived their RDA have established an average intake which will keep serum 25(OH)D concentration of a group above the 50 nmol/L target threshold ([6] and see below).

**Clarification** **#1.** Going forward there is a need to be more explicit in the definition of the RDA, and its equivalents, in particular on what is intended by the ‘average intake’, e.g., an average intake of an individual over a period of time (e.g., a week or longer). If this is the intended meaning, or if otherwise–it should be specified.

### 2.2. Serum 25(OH)D Thresholds Underpinning Dietary Requirement Estimates

Estimation of vitamin D intake requirement values, such as the EAR or RDA, is made more complex because they are established as usual intakes of vitamin D aimed to achieve specified serum 25(OH)D target concentrations in 50% or 97.5% of the population, respectively. The IOM indicated in 2011 that the data available to its *Vitamin D and Calcium* DRI committee did not lend themselves to use the standard process of DRI development, which is reliant on a normal distribution and the ability to determine an EAR intake value, and subsequently the RDA intake value, i.e., two standard deviations (SDs) above the EAR [6]. Instead, based on the availability of data, the committee used 25(OH)D concentrations to stimulate a dose–response relationship for vitamin D intake and bone health. This has also been a further source of divergence in the interpretation of the definition of the EAR and RDA. For example, NORDEN, following its review of the evidence base, decided that the physiological requirement for vitamin D in terms of bone health would mainly be met at a serum 25(OH)D concentration of 50 nmol/L [8]. Therefore, the EAR and RDA-equivalent recommendations they established (called AR and RI values, respectively) would maintain serum 25(OH)D concentrations over their specified 50 nmol/L in at least 50% and 97.5% of individuals, respectively [8].

In contrast, the IOM, who also used bone health outcomes as their chosen criterion, applied the conventional two SDs first and foremost to the serum 25(OH)D concentration underpinning the dietary requirement estimates [6]. For example, for adults the IOM suggested that a serum 25(OH)D concentration of 50 nmol/L would meet the needs of nearly all (97.5%) individuals, and assuming a normal distribution of requirements, a serum level of 40 nmol/L was set as being consistent with a median requirement (i.e., less two coefficients of variation (of 10% each), as a proxy for the two SDs). Of note, for children and adolescents, they did the reverse in which they suggested that a serum 25(OH)D concentration of 40 nmol/L covered the median requirement, and application of the two SDs brought this up to 50 nmol/L. The IOM coined the terms ‘EAR-like’ and ‘RDA-like’ for these underpinning serum 25(OH)D concentrations, to distinguish from EAR and RDA, respectively, which as the definitions dictate, are vitamin D intake values [6].

The EFSA panel who recently established their DRV for vitamin D in Europe, after taking into account the overall evidence and uncertainties for adults, infants and children, considered that there was sufficient evidence for an increased risk of adverse musculoskeletal health outcomes at serum 25(OH)D concentration below 50 nmol/L [7]. Thus, the panel concluded that a serum 25(OH)D concentration of 50 nmol/L could be used as a target value to derive a DRV for vitamin D intake for adults, infants, children and pregnant women. Of note, however, they did highlight that the setting and analyses of the available studies do not allow a conclusion to be drawn as to whether this concentration should be achieved by about half of (i.e., EAR-like) or most subjects (i.e., RDA-like) in the population [7].

**Clarification** **#2.** If serum 25(OH)D concentrations are to be used in future iterations of vitamin D DRV to underpin the establishment of EARs and RDAs, or their equivalents, it would be important to consider whether one concentration which is deemed to provide for sufficient vitamin D status should be selected (as per NORDEN [8]) or whether a concentration which is deemed to cover the median requirement should be used to which two SDs could be added to establish that covering the needs of 97.5% of individuals (as per IOM [6]).

### 2.3. From a Serum 25(OH)D Threshold to an EAR Intake Value for Vitamin D

**Figure 2:**
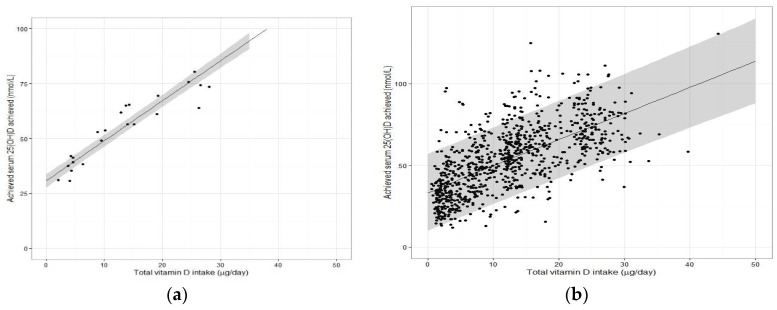
Relationship between total vitamin D intake and achieved serum 25(OH)D in winter time using data from seven randomized controlled trials (RCT) used in our recent individual participant data (IPD) level meta-regression [9]. (**a**) The black dots represent the aggregate RCT group mean data (*n* = 23 arms from the collection of 7 RCTs) with associated regression line and 95% confidence intervals around that mean response shown in gray shading. (**b**) The black dots represent the individual data point from each participant from the same 7 RCTs (*n* = 882 individuals) with associated regression line and 95% prediction intervals around that mean response shown in gray shading.

The step by which the ‘EAR-like’ and ‘RDA-like’ serum 25(OH)D concentrations are used to derive the EAR and RDA intake values, respectively, is potentially the one that has caused greatest confusion and is the one most in need of clarification as we move forward toward future iterations of DRV. Of less contention is the derivation of the EAR for vitamin D. As mentioned above, NORDEN established their AR of 7.5 μg/day for adult men and women from vitamin D intake-serum 25(OH)D dose-relationship data (see below) and using a serum 25(OH)D ≥50 nmol/L as indicative of sufficient vitamin D status [8]. The IOM also used vitamin D intake-serum 25(OH)D regression analyses, but selected a serum 25(OH)D concentration of 40 nmol/L, and established an EAR of 10 μg/day for those aged one year and upwards [6]. In both exercises, the regression analyses using group mean data from several winter-based vitamin D RCTs (referred to as the ‘standard meta-regression approach’) performed at northerly latitudes facilitated a derivation of an EAR estimate based on use of the median regression line (covering 50th percentile and underpinning an EAR) (see Figure 2a). As the collection of vitamin D RCTs used in the two exercises differed, we used the dataset from our recent meta-regression analysis [9] to illustrate the impact of the two different serum 25(OH)D target concentrations (40 versus 50 nmol/L) underpinning the EAR.

Using 50 nmol/L as the serum 25(OH)D threshold concentration, our EAR estimate of 8.8 μg/day (Table 2) is very similar to NORDEN’s AR of 7.5 μg/day, even though the collection of RCTs differed. These estimates should in theory maintain 50% of individuals with serum 25(OH)D above 50 nmol/L in winter (the other 50% could fall below this). In contrast, using the 40 nmol/L as the target threshold yielded our EAR estimate of 3.7 μg/day. IOM, using the 40 nmol/L, established a much higher EAR of 10 μg/day [6]. However, it is important to note that an intake of 10 μg/day in their regression modelling was associated with a predicted mean circulating 25(OH)D level of 59 nmol/L in children and adolescents, young and middle-aged adults. The DRI committee rationalised this by acknowledging the considerable uncertainty in the simulated dose-response relationship that needed to be taken into account, and accordingly selected the estimated intakes needed in a fashion that would err on the side of the specified intake “overshooting” the targeted serum value to ensure that the specified levels of intake achieved the desired serum 25(OH)D levels of 40 nmol/L [6].

### 2.4. From Serum 25(OH)D Thresholds into the RDA Intake Value

Beyond the EAR, clarity on the derivation of the RDA from the target serum 25(OH)D concentration is of key importance and in this step of the process, there has been much debate. Again, while NORDEN and IOM both based their RDA values based on the vitamin D intake-serum 25(OH)D dose-relationship data (and both used 50 nmol/L as the basis), the former established an intake of 10 μg/day [8], while the latter established 15 μg/day for those aged 1–70 (20 μg/day for those aged 71+ years) [6]. Before discussing the sources of confusion around how these estimates were derived, it might be worth re-emphasising how the RDA is used for assessment of nutrient intake.

The RDA intake value is set at a level that meets or exceeds the actual nutrient requirements of 97–98% of individuals in a given life stage and gender group. Thus, at this level of intake, there is a 2–3% probability of an individual not meeting his or her requirement (Figure 1b). The RDA has traditionally been adopted as the appropriate reference for planning intakes for individuals [4]. If one assumes a hypothetical individual X has undergone a dietary assessment, their practitioner works under the assumption that the RDA will achieve a serum 25(OH)D concentration of ≥50 nmol/L in this individual, irrespective of where this person actually resides within the distribution of requirements below the 97.5th percentile (see Figure 1). Thus, the RDA becomes the intake target in terms of dietary assessment as it will minimize the risk of inadequacy for that individual [4]. This is different entirely from suggesting that a group of individuals (e.g., population or population-subgroup) will be recommended/expected to consume the RDA. In a similar vein, the IOM, as a follow-up to their 2011 DRI report, went on to issue an announcement to clarify how the RDA for vitamin D was determined [10]. This statement highlighted that while the RDA, by definition, meets the requirements of 97.5% of the population, the goal is not, and should not be, to assure that 97.5% of the population exceeds the serum 25(OH)D value linked to the RDA (i.e., 50 nmol/L). They suggest doing so would shift the distribution to a higher level, and this could be associated with increased risk for potential adverse effects for some within the distribution [10]. 

In terms of the mechanics as to how the IOM and NORDEN established their RDA values, both used the lower 95% confidence interval (CI) of the median regression line of the vitamin D intake-serum 25(OH)D dose-relationship as a means of predicting the RDA intake (see Figure 2a for an example). As can be seen from Table 2, using the dataset from our meta-regression analysis [9] yielded an RDA estimate of 12.7 μg/day, not too dissimilar to that suggested by NORDEN (10 μg/day) or IOM (15 μg/day) for adults [6,8]. It should be noted again that the IOM estimate, as with the EAR estimate, erred on the side of caution in that the vitamin D intake of 15 μg/day would over-shoot the targeted serum 25(OH)D concentration of 50 nmol/L [6]. However, importantly we had indicated shortly after these IOM recommendations were issued, that if the expectation of the RDA intake estimate, derived by this standard meta-regression approach, is that it is an intake of vitamin D which is sufficient to cover the needs of 97.5% of individuals–it would fail to provide this level of protection [11]. The RDA (or equivalent) intake generated from the 95% CI of the median regression line represents an intake for which there is 95% surety it will maintain 50% of individuals over the target serum 25(OH)D concentration of 50 nmol/L. Thus, for example, if the above-mentioned individual X happens to have a need greater than that of the average person (i.e., 50 percentile), consuming 15 μg/day may not achieve a serum 25(OH)D concentration of 50 nmol/L. An intake that is capable of maintaining serum 25(OH)D concentration ≥50 nmol/L up at the 97.5th percentile of individuals, requires that it uses the median intake but plus 2 SDs beyond that, as per more conventional use of an RDA (Figure 1b). Deriving this latter estimate is not feasible at present from the type of standard meta-regression analyses that IOM or NORDEN have done of late because while such analyses, based on aggregate (group mean) data, perfectly captures the median response (and can add the 95% CI), they are not able to add the two required SDs, as information on the between-individual variability is not accessible [2,9,11]. Thus, while ideal for establishing an EAR, the standard meta-regression approach is not ideal for deriving an RDA.

### 2.5. Moving Away from Use of Aggregate Data from Vitamin D RCTs in Favour of Their Individual Data for the Estimation of the RDA for Vitamin D

**Figure 3:**
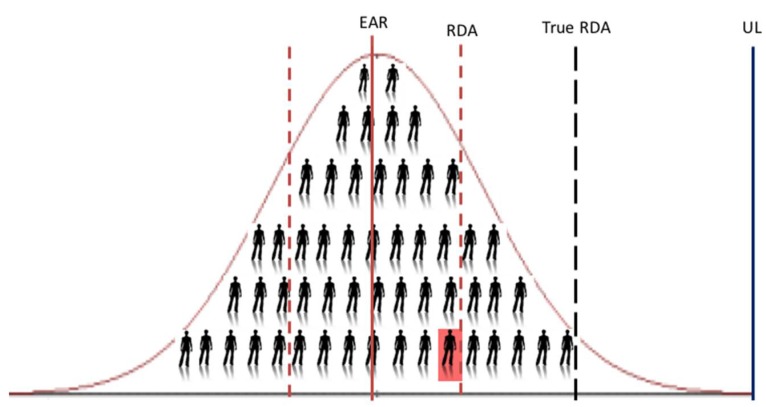
The placement of a hypothetical individual X (highlighted in red) close to the ‘RDA’ (as derived by use of the lower 95th confidence interval on the regression line), but much lower than the ‘True RDA’ is the estimated derived by use of the lower 95% prediction interval from the regression analysis. UL, Tolerable Upper Intake Level for vitamin D.

It is only when one uses the data from individuals within an RCT, or even better a collection of RCTs (see below), does the analyses allow one to capture the needed variability data and predict the intakes at the 97.5th percentile (mean + 2 SDs, i.e., the RDA). It does this by generating the lower 95% prediction interval from the data modelling (Figure 2b). This regression analysis based on individual participant data was the approach used by the Scientific Advisory Committee on Nutrition (SACN) in the UK for their recent age-group specific vitamin D DRVs [12]. The 95% prediction interval has been explained as an approximation of the interval that would allow for estimation of the requirements for 95% of individuals in the overall population [7]. Interestingly, the National Academy of Sciences in the US following a two-step exercise in which independent panels undertook a review process of suggested potential mathematical and statistical errors in the 2011 IOM report, clarified that the DRI committee did not use such a predication approach, but rather relied on the ‘overshoot’ arising from use of the 95% CI, mentioned above, to capture uncertainty [13]. Applying the lower 95% CI will help to overshoot these thresholds but will go no-where near replicating the requirement out at the 97.5th percentile, as we have demonstrated recently in a comparison of regression models using empirical data [9]. We showed that the usual vitamin D intake estimate needed to ensure a serum 25(OH)D ≥50 nmol/L up to and including the 97.5th percentile (using individual data, i.e., the ‘true RDA’ in Figure 3) is 30.9 μg/day, while that generated by use of the lower 95% CI of the median of the group mean response of the same dataset is 12.7 μg/day. This becomes very important when using these DRV to assess the vitamin D intake of an individual. For example, the IOM suggest that if an observed usual mean intake of an individual is between the EAR and the RDA, this probably needs to be improved (because the probability of adequacy is more than 50 percent but less than 97.5 percent) [4]. Only if intakes have been observed for a large number of days and are at or above the RDA should one have a high level of confidence that the intake is adequate [4]. Thus, using 12.7 μg/day versus 30.9 μg/day, as the true RDA will have a major impact.

Interestingly, the EFSA Panel considered that the available evidence did not allow the setting of ARs and PRIs for vitamin D and instead choose to set AIs for all population groups [7]. However, in setting their AIs, EFSA acknowledged the importance of using the lower 95% prediction interval approach in their meta-regression analyses [7]. EFSA using data from a large collection of vitamin D RCTs tried to include proxy data on between-individual variability within their meta-regression analyses by using data on serum 25(OH)D variability but from five population-based studies [14]. While this represents variability in serum 25(OH)D amongst individuals within a population, it is not the variability in response of serum 25(OH)D to increased vitamin D intake within individuals. This can be illustrated by their vitamin D intake estimate for 50 nmol/L at the 97.5th percentile (i.e., using their approach towards generating a lower 95% prediction interval) being only 16.1 μg/day [7].

**Clarification** **#3.** In terms of the RDA, there is a pressing need to revisit what we mean by the dietary intake level that is sufficient to meet the nutrient requirements of nearly all (97.5 percent) healthy individuals in a particular life stage and gender group, and specifically if we wish that such a value has the inherent confidence that it can cover the needs of all but 2–3% of such individuals. How this intake value is derived mathematically is also of key importance.

## 3. RCT Data Sharing Is Key to New DRVs for the Future

EFSA have suggested that one advantage of the standard meta-regression approach (as used by themselves, IOM and NORDEN) is the representativity, by considering several studies from various populations in different contexts, instead of relying on specific data from one specific study undertaken in a particular context [7], as was done by SACN for three age-groups [12]. However, and as discussed above, EFSA also indicated that by using group means from such studies, the information on the variability between individuals is diminished, which complicates the setting of an RDA [7]. Between-participant variability is crucial for estimating individual recommendations, such as the RDA [9].

To test the potential of such an approach, we recently undertook an IPD-based meta-regression using individual subject data from selected winter-based RCTs of the vitamin D intake–serum 25(OH)D dose-response [9]. A collection of seven, high-quality RCTs, where raw data were available to us, was included in the analysis (*n* = 883 individuals, ranging in age group from four years-olds to 65+ years), all implemented using the same study design, analytical platform for serum 25(OH)D and dietary assessment method [9]. These first IPD-derived estimates were shown to be considerably different from those of the agencies that used a standard meta-regression to analyze the vitamin D intake-serum 25(OH)D dose-response relationship, due to the inability of such standard meta-regression to capture between person-variability appropriately.

It has been stressed that IPD analyses are not without their challenges, including being resource intensive. The issue of limited availability of data for some studies could introduce bias [15]. Another option that has been suggested is to collaborate with other research groups and agree to pool resources to answer specific questions [16]. This is the approach adopted in our recent work which allowed us to secure serum samples for re-analysis of 25(OH)D and remove some of the method-related confounding that is likely intrinsic in DRV estimates to-date [9]. However, this could be considered the gold-standard approach and even if re-analysis of 25(OH)D is not feasible, the IPD using existing 25(OH)D data is still better than standard meta-regression analyses based on aggregate data. To underscore this notion, in the present work we took the data from our recent IPD versus standard meta-regression analyses, which was all based on standardized serum 25(OH)D data [9], and we applied the IPD analysis again but this time on the original non-standardized serum 25(OH)D data. As can be seen in Table 3, while the difference between the estimates from the standard meta-regression analyses based on aggregate data and that from the IPD analysis (both based on standardized serum 25(OH)D data) is significant, the estimates from the IPD analyses based on standardised and non-standardised 25(OH)D data are very similar, at least for this collection of RCTs.

Thus, gathering the serum 25(OH)D data from identified RCTs based on their original measurement methodology would suffice to undertake these IPD analyses, if standardisation of serum 25(OH)D data, as the gold standard, is not feasible. This may be important in a wider context, where the availability of samples in addition to data from RCTs identified in the systematic review component of the IPD meta-regression approach may not always be feasible and again could introduce bias.

The overall data sharing approach, however, may point towards an infrastructural requirement in that the instigator of such collaborative IPD analyses would need to be sufficiently expert in soliciting and handling the data as well as performing the regression modelling to derive the DRV estimates. This new approach may well benefit from the generation of formal guidelines detailing the steps needed. This might also raise awareness among potential collaborators of the need to also have good quality data on the ‘total vitamin D intake’ estimates (i.e., the summation of dietary vitamin D as well as assigned dose within the RCT) for all participants within their vitamin D RCTs. Going forward two important underpinning infrastructures which would enhance this aspect of IPD analyses is the standardisation of the assessment of habitual vitamin D intakes as well as standardisation of food composition data, as outlined previously [17].

This type of collaborative approach aligns with emerging drive towards using larger datasets, or ‘big data’, to help clinicians, policymakers, and the academic community evaluate an expanding evidence base with a view towards guidelines and recommendations. This IPD approach can also be tailored to different settings by altering the criteria for inclusion of the relevant vitamin D RCTs. For example, as more vitamin D RCTs have been published in children, pregnancy and ethnic population subgroups over recent years, tailored IPD analyses could be conducted to investigate the vitamin D dietary requirements for these specific subgroups.

Nutrient intake can pose a dual risk to health, due either to inadequate or excessive consumption [18]. While the benefits of the IPD-based approach are clear in terms of estimating the EAR and RDA to protect against vitamin D inadequacy, it also has an additional advantage in that its estimates can inform safety considerations. For example, while the lower prediction interval within the regression modelling yields estimates for the RDA, the higher prediction interval (Figure 2a) can be used to indicate the serum 25(OH)D concentrations achieved by the top 2.5th percentile of the population at that RDA intake and whether that concentration is in excess of 125 nmol/L, which the IOM suggest, if sustained, might be a reason for some concern [6]. Using our IPD-based ‘true’ RDA estimate of 28.8 μg/day (Table 3), the highest 2.5th percentile would achieve a serum 25(OH)D concentration of 110 nmol/L, and a vitamin D intake of ~38 μg/day would be needed before a serum 25(OH)D of 125 nmol/L would be breached by this 2.5th percentile (unpublished data). It should be noted, however, that the UL for vitamin D (i.e., the maximum level of total chronic daily intake of vitamin D (from all sources) judged to be unlikely to pose a risk of adverse health effects to humans) is the priority guidance in terms of safety of high vitamin D intakes [6,19]. While the RDA estimate from the IPD-based approach (~28 μg/day) is much closer to the UL than the RDA estimate derived from the standard aggregate-based method (see Figure 3), it is still well below the UL for vitamin D for children and adults, in the range 50–100 μg/day, depending on age-group, as established by EFSA [19] and the IOM [6].

Lastly, it is also important to emphasise that the IPD-based approach can be used under a variety of different thresholds of serum 25(OH)D. As mentioned above, much of the evidence-base underpinning the selection of a serum 25(OH)D threshold(s) in recent DRV exercises stemmed from (musculo)skeletal health outcomes. An emerging body of evidence for a causal relationship between vitamin D status and non-skeletal health outcomes could well lead to selection of a different serum 25(OH)D threshold in some future DRV exercise. As but one example to illustrate this point, a recent IPD of 25 eligible vitamin D supplementation RCTs (total 10,933 participants, aged 0 to 95 years) showed that vitamin D supplementation reduced the risk of acute respiratory tract infection by 12% among all participants [20], and a number of the ongoing vitamin D ‘mega-trials’ have respiratory disease as a pre-specified primary outcome measure, with these set to deliver their findings in the coming few years. Should these new data on the role of vitamin D status in respiratory health alter the serum 25(OH)D threshold selected to underpin an EAR and/or RDA, the IPD-based approach outlined in this review can just as easily be applied to this new threshold, as it has been applied by us using serum 25(OH)D thresholds which to-date have been on the basis of bone health outcomes.

## 4. How Food-Based Approaches Together with New DRV May Help Populations Improve Their Vitamin D Intake and Status

In terms of looking to the future, once some of the above-mentioned clarifications surrounding the DRV definitions and their interpretation/application are provided, and IPD meta-regression analyses is applied to the relevant vitamin D RCT data, we would have the most accurate DRV estimates of vitamin D requirements to-date. This would have clear public health protection benefits, beyond those from current DRV. While many, if not most, in the population will not have a dietary assessment, the application of these future DRV for population guidance on vitamin D intake and strategies to achieve the same, will also be of importance. For example, EFSA have suggested that the AR can be used to estimate the prevalence of inadequate intakes of micronutrients within a group (population) [5], if the distribution of nutrient intakes is normal, and intakes are independent from requirements. The percentage of the population with a habitual daily nutrient intake lower than the AR is taken as an estimate of the percentage of the population with probable inadequate intakes. For example, at a median intake equal to the AR, 50% of a population group will have intakes that may be inadequate for the chosen criterion of nutritional status [5]. Some of the panellists in the IOM DRI committee have suggested that given the inherent variability, the appropriate approach to achieve a low prevalence of vitamin D inadequacy within a population group—as verified by statistical modelling—is to shift the intake distribution so that most of the population (97.5%) have vitamin D intakes above the EAR of 10 μg/day (not above the RDA) [21].

Even using the existing dietary vitamin D requirement estimates from our recent IPD analyses [9], there seems to be a convergence of the different population status targets that would be attained should the vast majority of individuals achieve the EAR intake of 10 μg/day. For example, 97.5% of individuals would have serum 25(OH)D ≥25 nmol/L (as maybe the most conservative estimate of vitamin D deficiency, and that used by SACN in their DRV [12]), about 95% would have serum 25(OH)D ≥30 nmol/L (IOM, NORDEN and EFSA’s definition of vitamin D deficiency [6,7,8]), and about 50% of individuals would have serum 25(OH)D ≥50 nmol/L [9]. These IPD meta-regression projections are supported by recent population study-based findings. For example, the recent standardized serum 25(OH)D data from the Finnish Health 2011 study shows that in a representative sample of 4051 adults in Finland, a mean intake of 13 μg/day was associated with prevalence of serum 25(OH)D <30 nmol/L was <1% [22]. It should be acknowledged, however, that the very low prevalence in this population may have been aided by the fact that 63% of the sampling was done in August to October, when status would have been better (remainder in November to February). It is also worth noting that for most populations currently in Europe and North America, and likely beyond, between 50% and 100% of individuals fail to meet this EAR target of 10 μg/day [23,24,25,26]. Thus, in order for most of the population (97.5%) to achieve vitamin D intakes at or above 10 μg/day, strategic additions to the food chain will be required, as reviewed elsewhere [2,18,27,28,29].

## 5. Conclusions

It is important to highlight that the nutritional requirements for vitamin D that have emerged since 2011 were, for the most part, on the basis of (musculo) skeletal health outcomes [6,7,8,12,30]. Future iterations may well be based on different thresholds for 25(OH)D as scientific evidence accumulates and strengthens globally, and in particular as non-skeletal effects of vitamin D make an ongoing contribution to the discussion about thresholds for 25(OH)D that indicate a healthy vitamin D status. That said, the required clarifications to definitions of DRV and/or their interpretation and application as well as greater utilization and adoption of IPD meta-regression analyses will still pertain, thus these should be considered now in preparation for the next iteration of DRV for vitamin D. One of the information gaps and research needs as identified by the DRI committee in their 2011 report related to synthesizing evidence and research methodology, specifically the need to explore enhanced methodologies for data synthesis [6]. The IPD meta-regression approach outlined in this review is one such example of an enhanced methodology of key importance in terms of DRI development for vitamin D going forward. The application of such an approach will have even greater value moving forward when coupled with clarity of definition of some of the concepts guiding development of DRV for vitamin D.

## Figures and Tables

**Table 1:** Definitions for the constituent Dietary Reference Intakes and Dietary Reference Values.

Institute of Medicine’s *Dietary Reference Intakes* [4,6]	European Food Safety Authority’s *Dietary Reference Values* [5,7]
*Estimated Average Requirement (EAR*): The average daily nutrient intake level that is estimated to meet the requirements of half of the healthy individuals in a particular life stage and gender group.	*Average Requirement (AR):* The level of (nutrient) intake estimated to satisfy the physiological requirement or metabolic demand, as defined by the specified criterion for adequacy for that nutrient, in half of the people in a population group, given a normal distribution of requirement.
*Recommended Dietary Allowance (RDA):* The average daily dietary intake level that is sufficient to meet the nutrient requirements of nearly all (97.5 percent) healthy individuals in a particular life stage and gender group.	*Population Reference Intake (PRI)*: The level of (nutrient) intake that is adequate for virtually all people in a population group. On the assumption that the individual requirements for a nutrient are normally distributed within a population and the inter-individual variation is known, the PRI is calculated on the basis of the AR plus twice its standard deviation (SD). This will meet the requirements of 97.5% of the individuals in the population.
*Adequate Intake (AI):* The recommended average daily intake level of a nutrient based on observed or experimentally determined approximations or estimates of intakes that are assumed to be adequate for a group (or groups) of apparently healthy people; used when the RDA cannot be determined.	*Adequate Intake (AI):* The value estimated when a PRI cannot be established because an AR cannot be determined. An Adequate Intake is the average observed or experimentally determined approximations or estimates of nutrient intake by a population group (or groups) of apparently healthy people that is assumed to be adequate.
*Tolerable Upper Intake Level (UL):* The highest average daily nutrient intake level that is likely to pose no risk of adverse health effects to almost all individuals in the general population. As intake increases above the UL, the potential risk of adverse effects may increase.	*Tolerable upper intake level (UL):* The maximum level of total chronic daily intake of a nutrient (from all sources) judged to be unlikely to pose a risk of adverse health effects to humans.

**Table 2:** Comparison of the EAR and RDA estimates using two different serum 25(OH)D targets.

Serum 25(OH)D Concentration	EAR Estimate (μg/day) ^1^	RDA Estimate (μg/day) ^2^
40 nmol/L	3.7	-
50 nmol/L	8.8	12.7

^1^ Covering the vitamin D needs of 50% of individuals; ^2^ Covering the vitamin D needs of 97.5% of individuals.

**Table 3:** The RDA estimates for vitamin D as derived using different regression modelling approaches.

Regression Modelling Approach	RDA Estimate (μg/day) ^1^
Standard meta-regression (standardized 25(OH)D)	15.8
IPD meta-regression (standardized 25(OH)D)	28.8
IPD meta-regression (non-standardized 25(OH)D)	28.4

^1^ Covering the vitamin D needs of 97.5% of individuals.
